# Transformer-based deep learning for estimating bidirectional maternal-fetal cardiac coupling

**DOI:** 10.3389/fmedt.2026.1651162

**Published:** 2026-06-23

**Authors:** Murad Almadani, Leontios Hadjileontiadis, Ahsan Khandoker

**Affiliations:** 1 Healthcare Engineering Innovation Group, Department of Biomedical Engineering and Biotechnology, Khalifa University, Abu Dhabi, United Arab Emirates; 2 Department of Electrical and Computer Engineering, Aristotle University of Thessaloniki, Thessaloniki, Greece

**Keywords:** bidirectional coupling, deep learning, fetal ECG, fetal-maternal coupling, support vector machine, transformer

## Abstract

**Introduction:**

Maternal-fetal cardiac coupling has emerged as a promising non-invasive marker of fetal autonomic regulation and neurocardiac development. However, existing approaches are limited by unidirectional modeling, handcrafted coupling metrics, and a lack of interpretable and clinically validated abnormality assessment frameworks.

**Methods:**

We propose a transformer-based deep learning framework for bidirectional maternal-fetal cardiac coupling analysis using heart rate variability (HRV) features. The model learns fetal-to-maternal (FTM) and maternal-to-fetal (MTF) interactions using two independently trained UNETR-based models with identical architecture and no shared weights. The framework is evaluated on a real clinical dataset, incorporating time-delay, gestational, and classification analyses.

**Results:**

Time-delay analysis revealed directional asymmetry, with MTF coupling peaking at 3 seconds and FTM at 5 seconds. Gestational analysis showed relatively stable MTF coupling, while FTM coupling was consistently reduced in abnormal cases. Classification using support vector machines demonstrated strong discrimination, with the full-feature model (MTF + FTM + gestational week) achieving ROC-AUC = 0.91 ± 0.08. Under class imbalance, the coupling-only model (MTF + FTM) achieved the highest PR-AUC, indicating robust minority-class sensitivity.

**Discussion:**

These findings suggest that bidirectional coupling analysis may provide useful insight into maternal-fetal physiological interactions. However, the results should be interpreted as proof-of-concept evidence due to the limited and heterogeneous abnormal cohort. Further validation on larger and more homogeneous datasets is required to assess the potential of this approach for non-invasive prenatal monitoring.

## 1 Introduction

Maternal-fetal cardiac coupling refers to the temporal and physiological interaction between maternal and fetal heartbeats, offering crucial insights into fetal autonomic development and overall health. This coupling reflects the dynamic interplay between the maternal cardiovascular system and the developing fetal autonomic nervous system, particularly during key gestational periods (1, 2). Monitoring and quantifying this interaction can reveal vital physiological changes associated with fetal growth, maturation, and potential anomalies. Prior studies have demonstrated that maternal stress, respiration, physical activity, and sleep posture can significantly modulate fetal heart rate patterns through indirect pathways such as placental perfusion, hormonal regulation, and vagal tone modulation (3–5).

Traditional approaches to studying maternal-fetal coupling have primarily relied on indirect measurements of fetal heart rate (FHR) using ultrasound or Doppler techniques. While these methods are widely used in clinical settings, they lack the temporal resolution necessary to capture beat-to-beat variations. Non-invasive maternal abdominal ECG (maECG) has emerged as a promising alternative, enabling simultaneous recording of maternal and fetal cardiac signals. However, extracting fetal ECG from maECG remains technically challenging due to the dominance of maternal components and the presence of noise.

Advancements in signal processing and machine learning have significantly improved the extraction of fetal cardiac signals from maternal recordings (6–10). These methods have primarily focused on improving fetal ECG extraction and detecting cardiac peaks. Despite these advances, the direct study of maternal-fetal cardiac coupling, particularly the bidirectional temporal alignment between maternal and fetal beat-to-beat intervals, remains underexplored.

Conventional methods for evaluating maternal-fetal cardiac coupling often employ statistical techniques or phase synchronization analyses to assess the temporal relationship between maternal and fetal cardiac activities (11, 12). Other approaches employed methods such as phase locking (3), partial directed coherence (PDC) (11), transfer entropy (1, 13), additive autoregressive modeling (4), partial rank correlation (14), and bivariate phase-rectified averaging (15). While such methods provide valuable insights into coupling strength, they rely on static metrics, such as phase coherence or correlation, which lose their ability to dynamically capture the complex relationship between maternal and fetal cardiac systems (16, 17). A notable effort by (18) utilized maternal and fetal ECG signals in a deep learning framework to study coupling, but it was limited to a unidirectional analysis, missing the potential insights from bidirectional coupling.

In this study, we propose a novel transformer-based deep learning framework to analyze maternal-fetal cardiac coupling in a bidirectional manner. From maternal ECG and respiratory signals, our model predicts five key fetal HRV features, which are the mean R–R interval, the standard deviation of normal-to-normal intervals (SDNN), the root mean square of successive differences (RMSSD), the short-term variability (SD1), and the long-term variability (SD2). In the inverse direction, fetal ECG is used to predict the same maternal HRV features. Here, the maternal ECG originates from a thoracic lead and contains no fetal component, preventing fetal-origin information from entering the model. Also, the fetal ECG is extracted from abdominal data after maternal cancellation, and the predicted maternal HRV features are derived from an independent chest-lead channel. This configuration reduces the possibility of direct signal leakage and supports interpretation at the HRV interaction level, indicating that this bidirectional prediction framework enables a truly dynamic, data-driven assessment of maternal-fetal coupling strength during pregnancy. The results demonstrate the model’s high accuracy in estimating HRV features, highlighting its potential to uncover physiological interactions between maternal and fetal systems in a non-invasive manner.

Also, respiration is included in this study due to its physiological relevance to maternal–fetal interaction. Maternal respiratory cycles are known to modulate autonomic tone, vagal activity, and venous return, which can influence fetal heart rate variability through mechanical and hemodynamic pathways. Prior work has shown that respiratory-driven oscillations in maternal physiology can influence maternal–fetal heart rate relationships, with evidence of maternal respiratory activity affecting short epochs of cardiac synchronization between mother and fetus (3). In our framework, respiration is therefore used as an auxiliary signal in the maternal-to-fetal coupling direction to capture slow autonomic modulation rather than as a primary predictive variable.

In summary, this study makes the following contributions. To the best of our knowledge, we are the first to introduce a transformer-based bidirectional maternal–fetal cardiac coupling framework that models both directions using two direction-specific models built on the same UNETR architecture. Second, we formulate HRV-based coupling representation through a patch-based self-attention mechanism that enables multi-scale physiological dependency learning. Third, we demonstrate that bidirectional coupling provides complementary diagnostic information for abnormal fetal cardiac state detection, improving robustness over unidirectional coupling analysis under real clinical conditions.

This paper is organized as follows: Section 2 details the proposed methodology, including data preprocessing and model design. Section 3 presents experimental results focusing on the analysis of bidirectional coupling on both normal and abnormal cases. Section 4 discusses the implications of these findings, and Section 5 concludes with future directions.

## 2 Materials and methods

### 2.1 Dataset

To develop our transformer-based framework for maternal-fetal bidirectional cardiac coupling analysis, we initialize our deep learning training process using a synthetic dataset. Although the synthetic data refer to physiologically realistic and simulated ECG signals that mimic real-world data, it may not fully capture pathological conditions such as arrhythmias or structural abnormalities. Consequently, we used a real clinical dataset for model fine-tuning, which includes actual recordings from pregnant women, covering both normal and pathological cases.

#### 2.1.1 Synthetic training dataset

For model pre-training, we used the FECGSYN dataset from PhysioNet (19, 20), which provides realistic simulations of maternal and fetal ECG signals. This dataset is widely adopted in fetal ECG research due to its physiological consistency with real clinical data. It includes recordings from 10 subjects, each containing five minutes of maternal and fetal ECG signals sampled at 250 Hz. To ensure effective training and consistent with ultra-short-term HRV analysis, we segmented each recording into 10 s windows, which have been shown to reliably estimate time-domain HRV metrics such as RMSSD and SDNN (21, 22). The following HRV features were computed for each segment:

- Mean R-R interval
- Standard deviation of R-R intervals (SDNN)
- Root mean square of successive differences (RMSSD)
- Short-term and long-term variability

Additionally, maternal respiratory signals were derived by applying a low-pass filter to an abdominal ECG lead near the chest. These extracted features served as model inputs for learning maternal-to-fetal (MTF) and fetal-to-maternal (FTM) coupling dynamics.

It is important to note that FECGSYN is used solely to initialize the encoder with physiologically plausible ECG morphology and temporal dynamics, rather than to model maternal–fetal coupling. Coupling behavior is calculated exclusively after fine-tuning on the real clinical dataset, which will be discussed next.

#### 2.1.2 Real clinical dataset for fine-tuning and testing

After pre-training on synthetic data, we fine-tuned and evaluated our model using a real-world dataset collected from 85 pregnant subjects (70 normal and 15 abnormal cases). Normal data were collected during gestational weeks 17 to 40 over 10 min clinical sessions, while abnormal cases span gestational weeks between 18 and 37 with a recording length of 1 minute. The demographics summary of the included abnormal cases is depicted in Table 1. Ethical approval for the collection and use of the clinical dataset was obtained through Tohoku University Institutional Review Board protocols as detailed in previously published studies (23). This real clinical dataset is a proprietary dataset previously collected under institutional ethical approval (Approval number: 2021-1-133).

**Table 1:** Abnormal demographics summary including gestational and maternal height and weight, and identified abnormalities. The gestational age ranges between 18–37 weeks.

Patient ID	Gestational week	Height	Weight	Abnormality
1	34	–	–	Heart anomaly
2	30	–	–	Heart failure
3	24	162 cm	95 kg	Placental dysfunction
4	23	–	–	VSD, ASD, CDH, Chromosomal aberration
5	36	164 cm	68 kg	Heart anomaly
6	18	–	–	TTTS donor
7	31	–	–	Medical history of intrauterine fetal death
8	33	164 cm	68 kg	Fetal tachycardia
9	35	165 cm	63 kg	WPW
10	29	–	–	NIHF
11	35	166 cm	63.3 kg	Fetal tachycardia
12	28	–	–	Fetal tetralogy of Fallot, VSD, PA, MS, PAC
13	26	–	–	Ebstein
14	27	–	–	AV block, CHD, SA, CAV
15	37	–	–	IUGR

VSD, Ventricular Septal Defect; ASD, Atrial Septal Defect; CDH, Congenital Diaphragmatic Hernia; TTTS, Twin-to-Twin Transfusion Syndrome; WPW, Wolff-Parkinson-White syndrome; NIHF, Non-immune Hydrops Fetalis; PA, Pulmonary Atresia; MS, Mitral Stenosis; PAC, Premature Atrial Contraction; AV block, Atrioventricular Block; CHD, Congenital Heart Disease; SA, Single Atrium; CAV, Cardiac Allograft Vasculopathy; IUGR, Intrauterine Growth Restriction; CAC, Common Atrioventricular Canal.

Eleven custom-made gold electrodes with silver chloride gel were placed on the abdomen, a reference electrode on the back, and one on the chest to capture maternal ECG. This standardized setup ensured consistent and reliable signal acquisition across participants. Fetal ECG extraction was performed by incorporating maternal ECG cancellation and blind source separation techniques (2, 24). This method enabled precise detection of fetal R-peaks despite maternal signal interference.

Similar to the synthetic dataset, the real ECG recordings were segmented into 10 s intervals to maintain consistency in feature extraction. The same set of HRV features, including mean R-R interval, SDNN, RMSSD, and short/long-term variability, were computed for both maternal and fetal signals. Maternal respiratory signals were also extracted through a 4th-order low-pass Butterworth filter with a 0.4 Hz cutoff. Since maternal ECG energy lies primarily above 1 Hz, this operation removes the ECG band by design, leaving a slow-varying signal dominated by respiratory and autonomic trends. For the MTF coupling analysis, maternal ECG and respiratory signals were used to predict fetal HRV features. Likewise, fetal ECG was used to estimate maternal HRV features for FTM coupling direction. This dataset enabled a bidirectional assessment of maternal-fetal cardiac interactions, providing a foundation for understanding adaptive physiological mechanisms throughout gestation.

### 2.2 Proposed deep pipeline

**Figure 1:**
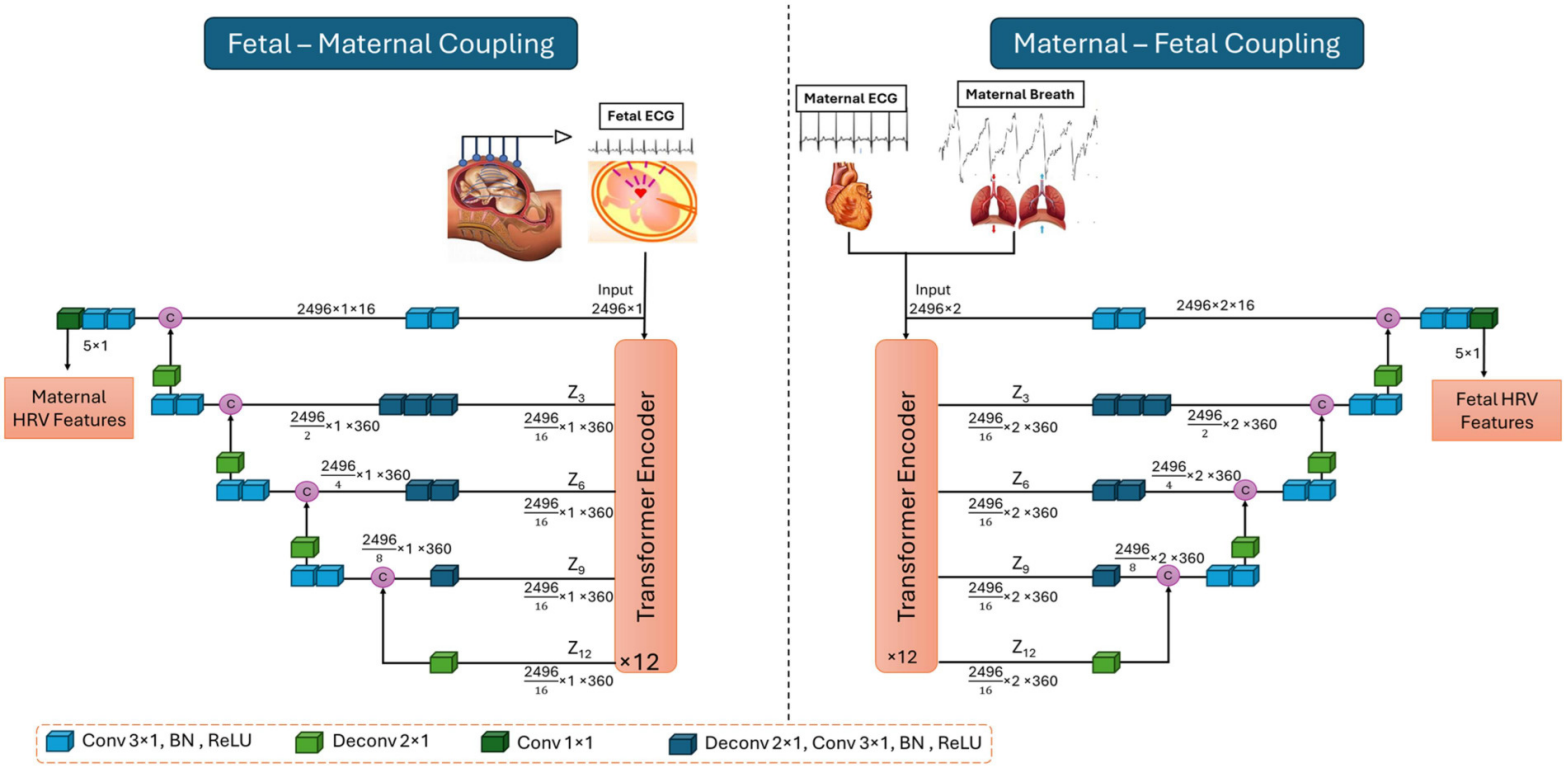
Illustration of the proposed transformer-based pipeline for bidirectional maternal-fetal cardiac coupling. The right pipeline represents maternal-to-fetal (MTF) coupling, where 10 s of maternal ECG and respiratory signals are processed through convolutional layers and a transformer to estimate fetal HRV features. The left pipeline depicts fetal-to-maternal (FTM) coupling, using 10 s of fetal ECG to predict maternal HRV features through a similar architecture. Here, C denotes channel-wise feature concatenation between upsampled decoder features and the corresponding transformer skip connections.

The proposed deep learning pipeline is designed to analyze bidirectional maternal–fetal cardiac coupling by learning temporal and physiological dependencies in both fetal-to-maternal (FTM) and maternal-to-fetal (MTF) directions. The architecture consists of two independent symmetric processing streams based on the same one-dimensional UNETR backbone with a transformer encoder proposed in (6), as illustrated in Figure 1. Both branches are trained and evaluated independently, but share identical encoder, transformer, and decoder structures and differ only in their input modalities and prediction targets. The original UNETR formulation is adapted below from waveform reconstruction to HRV feature regression while preserving the patch-based transformer encoder and multi-scale skip-connected decoder structure.

#### 2.2.1 UNETR

When dealing with one-dimensional physiological input signals, a transformer-based UNETR architecture (6) is adopted. A one-dimensional input signal $x\in {ℜ}^{T\times 1\times C}$ is first formed, where T denotes the temporal length of the 10 s segment and C denotes the number of input channels (C=1 for fetal ECG in the FTM direction and C=2 for maternal ECG and respiration in the MTF direction). The input signal is partitioned into non-overlapping patches with size N=16. A linear layer projects each patch into a K-dimensional transformer embedding space, where K=360 in our system.

To preserve the temporal positional information contained in the recovered patches, a positional embedding $E_{\mathrm{pos}}\in {ℜ}^{N\times K}$ is added to the embedded projections $E\in {ℜ}^{T\times K}$, as shown in the following equation (1):

$$z_{0}=[x_{v}^{1}E;x_{v}^{2}E;\cdots ;x_{v}^{N}E]+E_{\mathrm{pos}}.$$ (1)

The transformer consists of stacked blocks that contain multi-head self-attention (MSA) and multilayer perceptron (MLP) sub-layers, which is defined in the equation (2) below:

$$z_{i}^{{}^{\prime }}=\mathrm{MSA}(\mathrm{Norm}(z_{i-1}))+z_{i-1},\;i=1\cdots .L$$ (2)

and in the following equation (3):

$$z_{i}=\mathrm{MLP}(\mathrm{Norm}(z_{i}^{{}^{\prime }}))+z_{i}^{{}^{\prime }},\;i=1\cdots .L$$ (3)

where i denotes the transformer block index and L=12 is the number of transformer layers. A normalization operation is applied before each MSA and MLP block to ensure stable feature scaling across layers. The MLP consists of two fully connected hidden layers activated by GELU functions. Each MSA block contains 12 parallel self-attention (SA) heads, that is defined in the equation (4):

SA(z)=Av. (4)

The self-attention weights are computed using the query (**q**), key (**k**), and value (**v**) representations in the embedded sequence $z\in {ℜ}^{N\times K}$ , that is defined in equation (5).

$$A=\mathrm{Softmax}\left(\frac{qk^{T}}{\sqrt{K_{h}}}\right),$$ (5)

Here, the scaling factor $K_{h}=K/n$ preserves parameter stability across the n=12 attention heads. The final multi-head self-attention output is shown in the following equation (6):

$$\mathrm{MSA}(z)=[{\mathrm{SA}}_{1}(z);{\mathrm{SA}}_{2}(z);\cdots ;{\mathrm{SA}}_{n}(z)]W_{\mathrm{msa}}$$ (6)

where $W_{\mathrm{msa}}\in {ℜ}^{K\times K}$ denotes the learnable projection matrix.

Inspired by U-Net-like architectures, the intermediate transformer outputs $z_{i}$ at depths i∈{3,6,9,12} are extracted with shape $\frac{T}{16}\times K$. These representations are reshaped and projected from the embedding space back into the signal space using consecutive 3×1 convolutional layers followed by normalization, forming multi-scale skip connections to the decoder.

At the bottleneck of the encoder (i.e., the final transformer layer), deconvolution is applied to increase the feature resolution by a factor of two. The decoder progressively upsamples the latent feature maps using deconvolutionary layers followed by three 3×1 convolutional layers. At each stage, the upsampled features are concatenated with the corresponding transformer skip features (e.g., $z_{9}$, $z_{6}$, $z_{3}$) for refined multi-scale reconstruction.

In contrast to the segmentation setting of the original UNETR model, the final decoder output in this work is passed through a fully connected regression head that maps the latent representation into a five-dimensional HRV feature vector (mean R–R interval, SDNN, RMSSD, SD1, and SD2), rather than using a 1×1 convolution and softmax activation.

#### 2.2.2 Fetal-to-maternal coupling

The FTM coupling architecture predicts maternal heart rate variability (HRV) features from fetal ECG input using the same one-dimensional UNETR backbone previously discussed. Each 10 s fetal ECG segment is processed through the patch-based transformer encoder and multi-scale decoder with skip connections extracted from intermediate transformer layers $z_{3}$, $z_{6}$, $z_{9}$, and $z_{12}$. The core of the architecture is a 12-layer transformer encoder that models long-range temporal dependencies in the embedded feature space. The decoder subsequently reconstructs the maternal HRV feature vector from the latent representation using progressive deconvolution and convolutional refinement stages, followed by a fully connected regression head.

#### 2.2.3 Maternal-to-fetal coupling

The MTF coupling architecture follows the same UNETR-based encoder–transformer–decoder pipeline described above, but with dual-channel input. The maternal ECG and the corresponding low-pass filtered respiratory signal are concatenated channel-wise and provided as a two-channel input to the shared UNETR encoder. The stacked signal is processed through the same patch embedding, transformer encoder, and multi-scale skip-connected decoder to estimate the fetal HRV feature vector. Thus, both coupling directions rely on an identical deep backbone and differ only in their input configuration and regression targets.

#### 2.2.4 Implementation details

To prevent data leakage and ensure unbiased abnormality classification, all model development stages were performed using strict subject-level separation. Specifically, Leave-One-Subject-Out (LOSO) (25) cross-validation was conducted throughout the study. In each fold, the deep UNETR model was trained exclusively on the training subjects and subsequently used to compute DCS features only for the held-out test subject. The support vector machine classifier was then applied to the DCS values derived from the unseen test subject. No subject contributed to both training and testing within any fold, ensuring complete separation between representation learning and evaluation.

The proposed model was implemented using the PyTorch and MONAI frameworks and trained on an NVIDIA RTX 3060 GPU with 6 GB of memory. To improve robustness to outliers in HRV estimation, the Huber loss function was employed instead of the conventional mean squared error (26). The Huber loss function is shown in the following equation (7):

$$L_{\text{Huber}}(y,\hat{y})=\left\{ \begin{array}{cc} \frac{1}{2}(y-\hat{y}{)}^{2},\; & \text{if}\;|y-\hat{y}|\leq \delta \;\\ \delta |y-\hat{y}|-\frac{1}{2}\delta^{2},\; & \text{otherwise}\; \end{array}\right.$$ (7)

where y is the ground truth HRV feature, $\hat{y}$ is the predicted output, and δ is set to 1.

Optimization was performed using the Adam optimizer (27) with an initial learning rate set to 0.0001. Following the UNETR configuration described previously, the transformer encoder operates on non-overlapping 1D patches of length N=16 with an embedding dimension of K=360. The transformer consists of L=12 stacked encoder layers with 12 self-attention heads per layer. Learnable positional embeddings are added to the patch embeddings prior to transformer processing. The same UNETR backbone architecture is used for both the FTM and MTF branches; however, the two models are trained independently and do not share parameters. In contrast to the segmentation setting of the original W-NETR model, the final layer in this work is a fully connected regression head that outputs five HRV features.

### 2.3 Deep coupling strength

The concept of deep coupling strength (DCS) quantifies the bidirectional interactions between maternal and fetal cardiac systems. It is derived from the outputs of the proposed deep pipeline, which estimates maternal and fetal heart rate variability (HRV) features. The DCS is a measure of how strongly these physiological systems are interconnected, offering insight into their adaptive mechanisms.

For each direction of coupling (FTM and MTF) the predicted HRV features are compared to the ground truth using the Mean Absolute Percentage Error (MAPE) Complement. This comparison captures the degree of alignment between the modeled and actual HRV dynamics. The deep coupling strength is defined as the normalized similarity between the two signals, which is calculated using the following equation (8):

DCS=1−MAPE (8)

where MAPE is defined in equation (9):

$$\mathrm{MAPE}=\frac{\sum_{n=1}^{N}\frac{\left|\mathrm{GT}-\mathrm{Prediction}\right|}{\mathrm{GT}}}{N}$$ (9)

and the GT refers to the ground truth.

Here, it should be noted that DCS is computed on physiologically derived HRV feature trajectories rather than on raw signals, and is used as a comparative indicator of coupling behavior across directions and conditions rather than as an absolute prediction accuracy metric, such that higher estimation accuracy indicates stronger coupling, reflecting the model’s ability to capture the physiological relationship between maternal and fetal cardiac systems. This interpretable, data-driven metric enables a robust analysis of bidirectional coupling dynamics, highlighting gestational changes and potential abnormalities in cardiac interactions.

Importantly, DCS should not be interpreted as a generic regression performance metric. In this framework, the prediction target (e.g., fetal HRV features in the MTF direction) is generated exclusively from signals originating in the opposite physiological system (e.g., maternal ECG and respiration). Therefore, accurate prediction is only possible if statistically and temporally meaningful cross-system dependencies exist. If maternal and fetal HRV dynamics were independent, the model would converge toward population averages and yield low DCS values. Thus, DCS operationalizes coupling as cross-system predictive dependency rather than raw signal similarity.

To further distinguish DCS from trivial model fitting, we evaluated coupling strength across varying temporal delays (Section 3.1). The presence of distinct non-zero delay peaks, with directional asymmetry (MTF ≈ 3 s, FTM ≈ 5 s), supports the interpretation that DCS captures physiologically plausible time-lagged interactions rather than instantaneous or circular inference. Moreover, the systematic reduction of FTM coupling in abnormal pregnancies further suggests that DCS reflects underlying physiological integrity rather than purely statistical fit.

To prevent bias arising from unequal recording durations (10 min for normal cases vs. 1 min for abnormal cases), DCS was first computed at the window level and subsequently aggregated per subject by averaging across all windows belonging to that subject. All downstream statistical analyses and classification experiments were performed using subject-level aggregated DCS values rather than per-window values. This ensures that each subject contributes equally to the analysis regardless of recording length.

## 3 Results

### 3.1 Temporal delay in coupling dynamics

**Figure 2:**
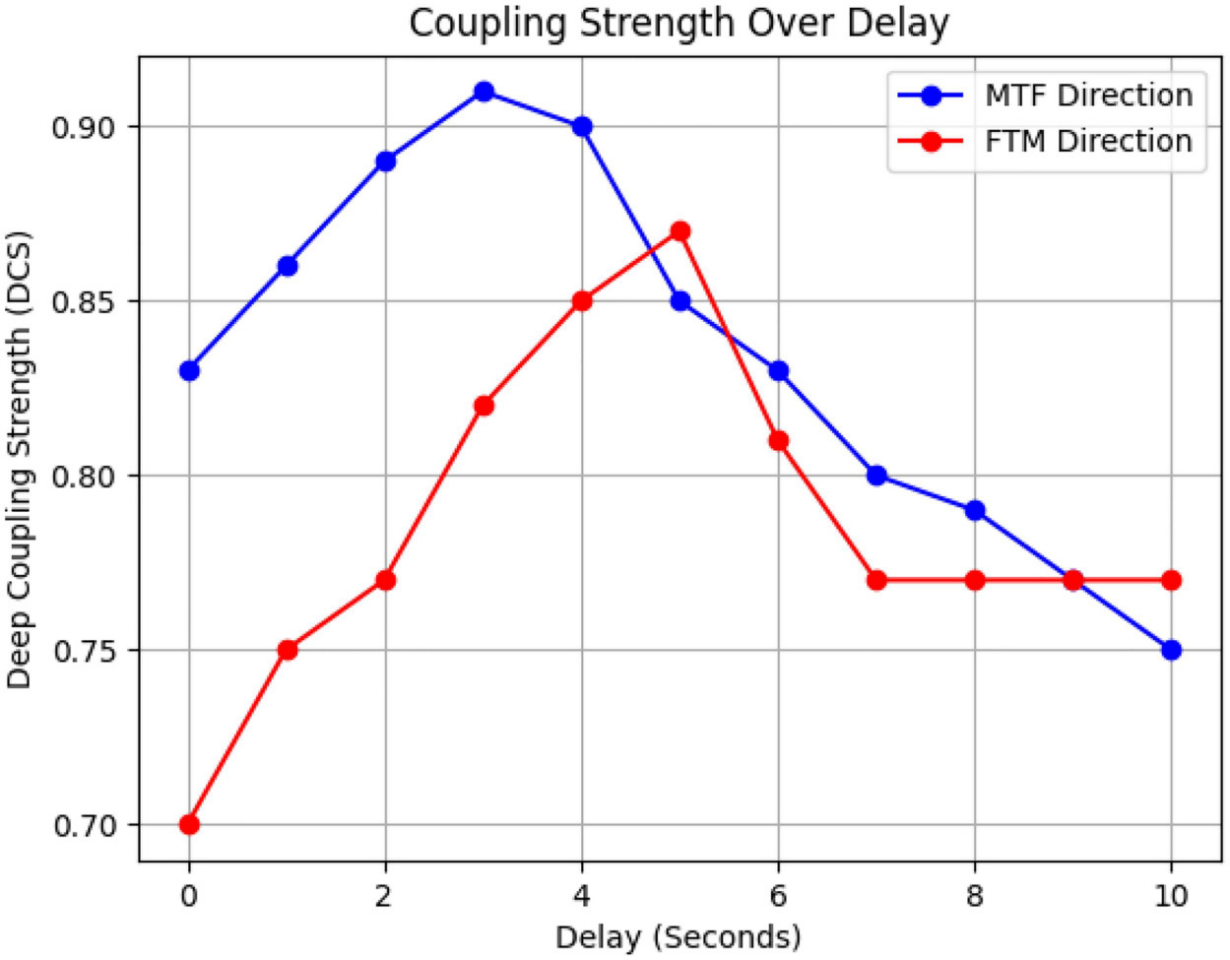
Coupling Strength Over Delay: Mean accuracy of MTF and FTM coupling strengths across varying time delays.

To characterize the temporal nature of maternal-fetal cardiac interactions, we conducted a delay-based analysis by systematically introducing lags of 0 to 10 s between input and output signals for both MTF and FTM coupling pathways. As depicted in Figure 2, coupling strength exhibits distinct peaks at specific delays for each direction.

The MTF coupling strength reaches its maximum at a delay of approximately 3 s, indicating that maternal heart activity precedes and influences fetal responses with a short lag. Conversely, FTM coupling peaks at around 5 s, suggesting a more delayed impact of fetal activity on the maternal heart. These results illustrate the directional asymmetry and time-dependent nature of the interaction, underscoring the importance of incorporating temporal lag in models of maternal-fetal physiological coupling.

### 3.2 Coupling behavior in normal pregnancies

**Figure 3:**
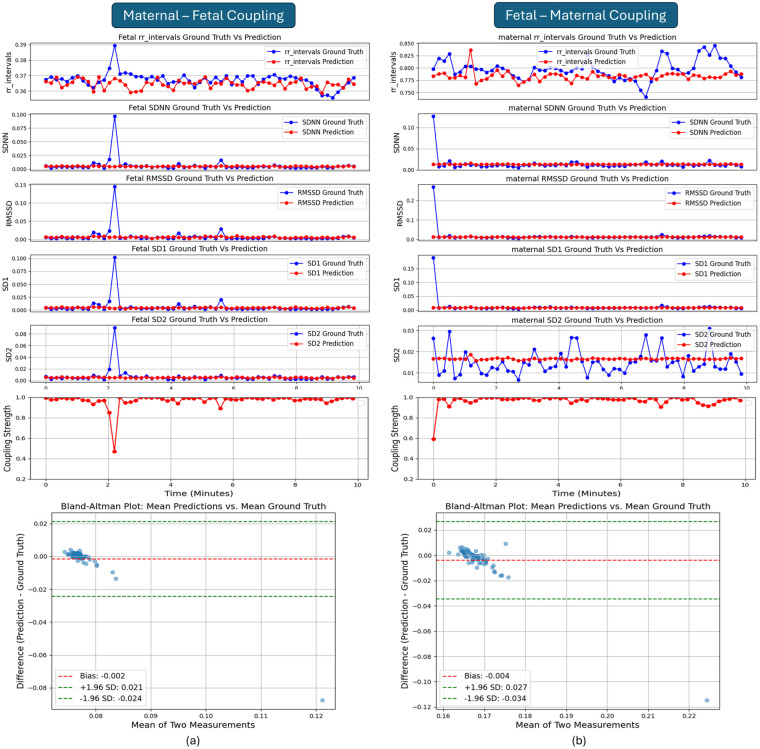
Comparison of ground truth and predicted HRV features for MTF (fetal HRV; **a**) and FTM (maternal HRV; **b**) coupling analysis in a representative normal subject. Top panels show predicted and actual HRV features over time, with calculated coupling strength indicating periods of strong and weak coupling. Bland-Altman plots below each figure assess agreement between predicted and actual mean HRV values.

To validate model performance under normal physiological conditions, we assessed the predicted vs. ground truth HRV features for both MTF and FTM coupling. Figure 3 presents example results from a 28-week gestation case, where high coupling strength aligns well with accurate prediction of HRV features. The top panels illustrate temporal alignment between model outputs and reference values, while the Bland-Altman plots below show minimal bias and narrow agreement limits, confirming the model’s accuracy in estimating maternal-fetal cardiac coupling during typical development.

### 3.3 Coupling behavior in pathological pregnancies

**Figure 4:**
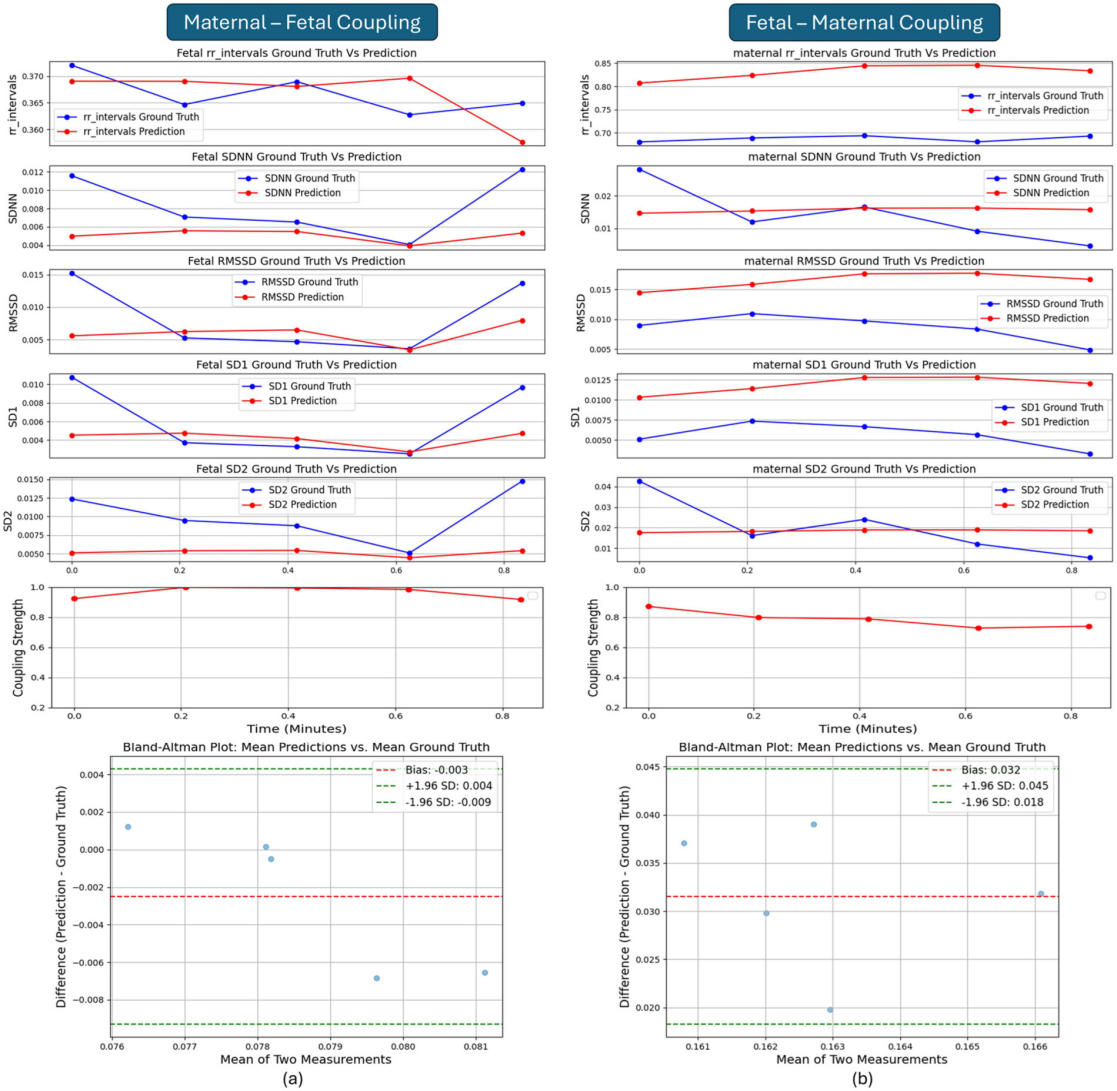
Comparison of ground truth and predicted HRV features for MTF (fetal HRV; **a**) and FTM (maternal HRV; **b**) coupling analysis in an IUGR case. Top panels show predicted and actual HRV features over time, with calculated coupling strength indicating periods of strong and weak coupling. Bland-Altman plots below each figure assess agreement between predicted and actual mean HRV values.

We further evaluated the model on a pathological case involving intrauterine growth restriction (IUGR) at 37 weeks gestation. Figure 4 illustrates both MTF and FTM coupling predictions. The model maintains high accuracy for MTF coupling, effectively capturing maternal influence despite abnormal conditions. In contrast, FTM coupling exhibits lower accuracy, possibly reflecting reduced fetal influence due to compromised physiology. This pattern is supported by the corresponding Bland-Altman analysis, which reveals weaker agreement for FTM estimates. These findings suggest that impaired fetal-maternal coupling may serve as a physiological indicator of stress or abnormality, suggesting potential relevance for identifying abnormal physiological patterns in high-risk pregnancies.

### 3.4 Coupling analysis over fetal development

**Figure 5:**
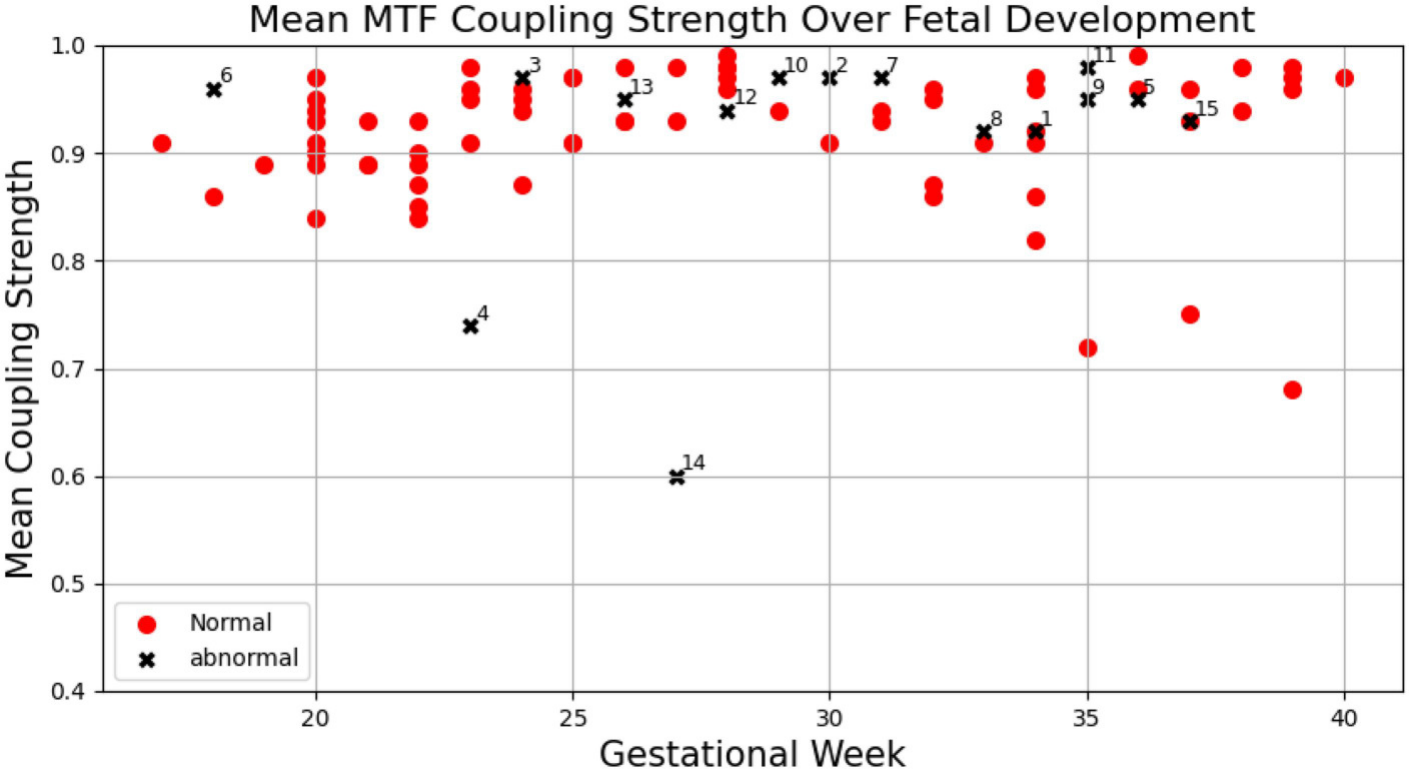
Mean MTF coupling strength from gestational week 17 to 40. Red circles denote normal pregnancies, while black crosses indicate abnormal cases. Patient IDs for abnormal samples are labeled above each respective point.

We explored the developmental progression of bidirectional coupling across gestation. Figure 5 shows that MTF coupling in abnormal cases generally mirrors the trend observed in normal cases, limiting its ability to differentiate between the two groups solely based on mean coupling strength.

**Figure 6:**
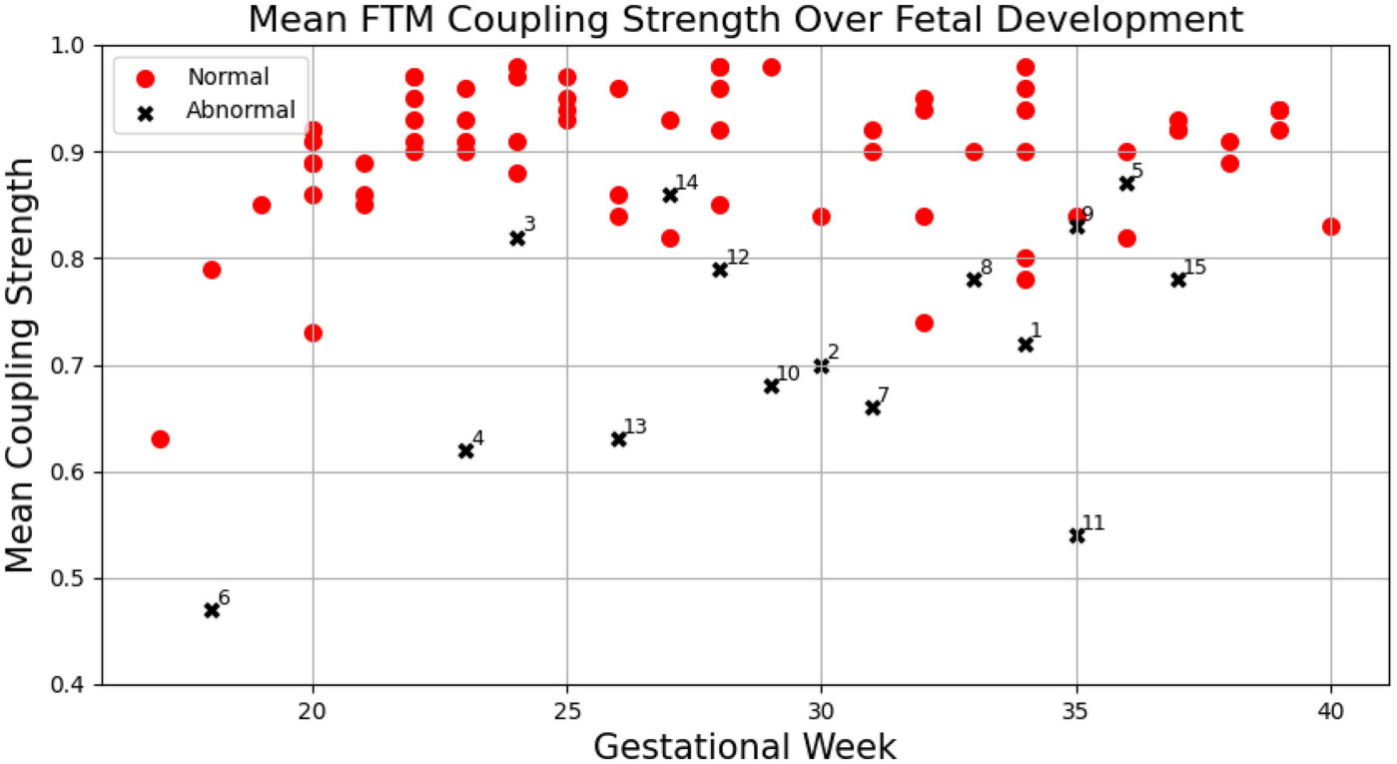
Mean FTM coupling strength from gestational week 17 to 40. Normal cases are marked in red, and abnormal cases are shown as black crosses with corresponding patient IDs annotated.

In contrast, Figure 6 reveals that FTM coupling is notably reduced in abnormal pregnancies, reflecting disrupted or weakened fetal influence on maternal cardiac dynamics. This distinction highlights the utility of FTM coupling strength as a more sensitive indicator of physiological compromise and potential fetal distress.

### 3.5 Cohort-level HRV regression performance

To further characterize the regression performance of the proposed bidirectional framework, we analyzed prediction errors for each individual HRV feature at the cohort level. For every subject, the mean absolute error (MAE) and root mean square error (RMSE) between predicted and ground truth HRV features were computed and then summarized across subjects for both maternal-to-fetal (MTF) and fetal-to-maternal (FTM) coupling directions.

**Figure 7:**
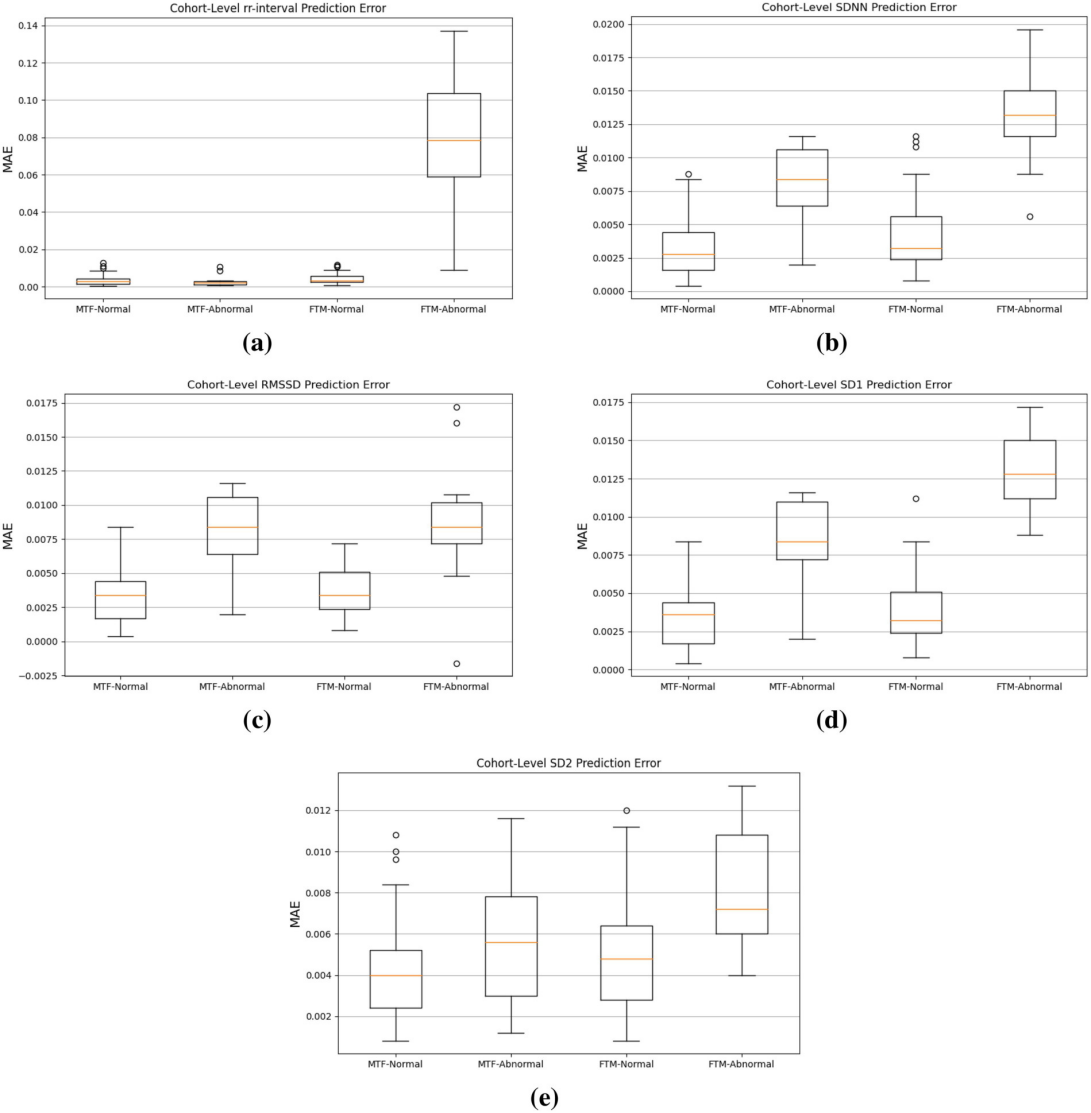
Cohort-level regression performance for individual HRV features using mean absolute error (MAE). Each boxplot summarizes subject-level prediction errors for maternal-to-fetal (MTF) and fetal-to-maternal (FTM) coupling directions for both normal and abnormal pregnancies. (**a**) Mean R-R interval, (**b**) SDNN, (**c**) RMSSD, (**d**) SD1, and (**e**) SD2.

Figure 7 and Table 2 present the distribution of subject-level MAE and RMSE values for the five HRV features considered in this study: mean R–R interval, SDNN, RMSSD, SD1, and SD2. Results are shown separately for normal and abnormal pregnancies to highlight potential directional differences in predictive performance.

**Table 2:** Cohort-level RMSE values (mean ± standard deviation) for each HRV feature and coupling direction.

Feature	MTF-normal	MTF-abnormal	FTM-normal	FTM-abnormal
RR interval	0.0035±0.0018	0.0028±0.0010	0.0048±0.0025	0.0910±0.0320
SDNN	0.0036±0.0019	0.0097±0.0030	0.0045±0.0023	0.0142±0.0038
RMSSD	0.0042±0.0020	0.0091±0.0030	0.0040±0.0018	0.0100±0.0038
SD1	0.0044±0.0021	0.0095±0.0033	0.0044±0.0020	0.0142±0.0039
SD2	0.0050±0.0022	0.0068±0.0027	0.0060±0.0028	0.0088±0.0030

Across all HRV features, the MTF direction consistently demonstrates low prediction error with tightly distributed MAE and RMSE values for both normal and abnormal pregnancies. This indicates that maternal physiological signals provide stable predictive information for estimating fetal HRV dynamics across gestational conditions.

In contrast, the FTM direction exhibits noticeably larger prediction errors in abnormal pregnancies across all HRV metrics. While FTM prediction errors remain relatively small in normal cases, abnormal pregnancies show a systematic increase in MAE and RMSE for mean R–R interval as well as variability-based features (SDNN, RMSSD, SD1, and SD2). This consistent degradation across multiple HRV measures suggests a reduction in the predictive influence of fetal cardiac dynamics on maternal HRV under pathological conditions.

Importantly, the directional asymmetry observed in these regression results aligns with the coupling-strength analysis reported earlier. The preserved MTF prediction accuracy together with the elevated FTM error in abnormal cases supports the interpretation that fetal-to-maternal coupling becomes weakened or disrupted when fetal physiological regulation is compromised. Therefore, the regression analysis further substantiates the physiological interpretation of deep coupling strength as a measure of cross-system predictive dependency rather than merely a reflection of model fitting accuracy.

**Figure 8:**
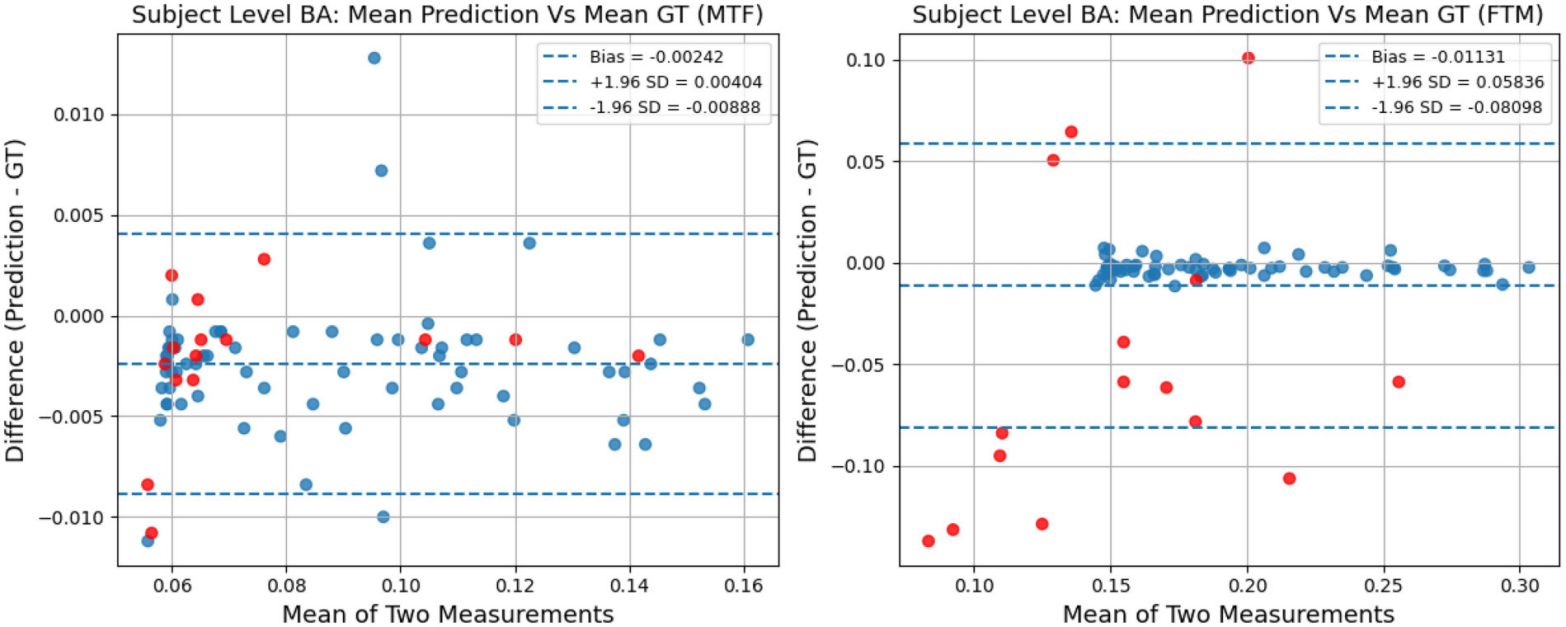
Subject-level Bland–Altman analysis for the mean features prediction in the maternal-to-fetal (left) and fetal-to-maternal (right) directions. Each point represents one subject. Abnormal cases are highlighted in red.

To further assess the agreement between the predicted and ground-truth of the mean feature values at the subject level, Bland–Altman plots were generated for both the MTF and FTM directions, as shown in Figure 8. Notably, many of the abnormal subjects, highlighted in red, deviated more clearly from the main cluster, especially in the FTM plot. This observation is consistent with the overall regression results, where the FTM direction was more strongly affected by abnormal cases than the MTF direction.

### 3.6 Abnormality detection using coupling analysis

**Figure 9:**
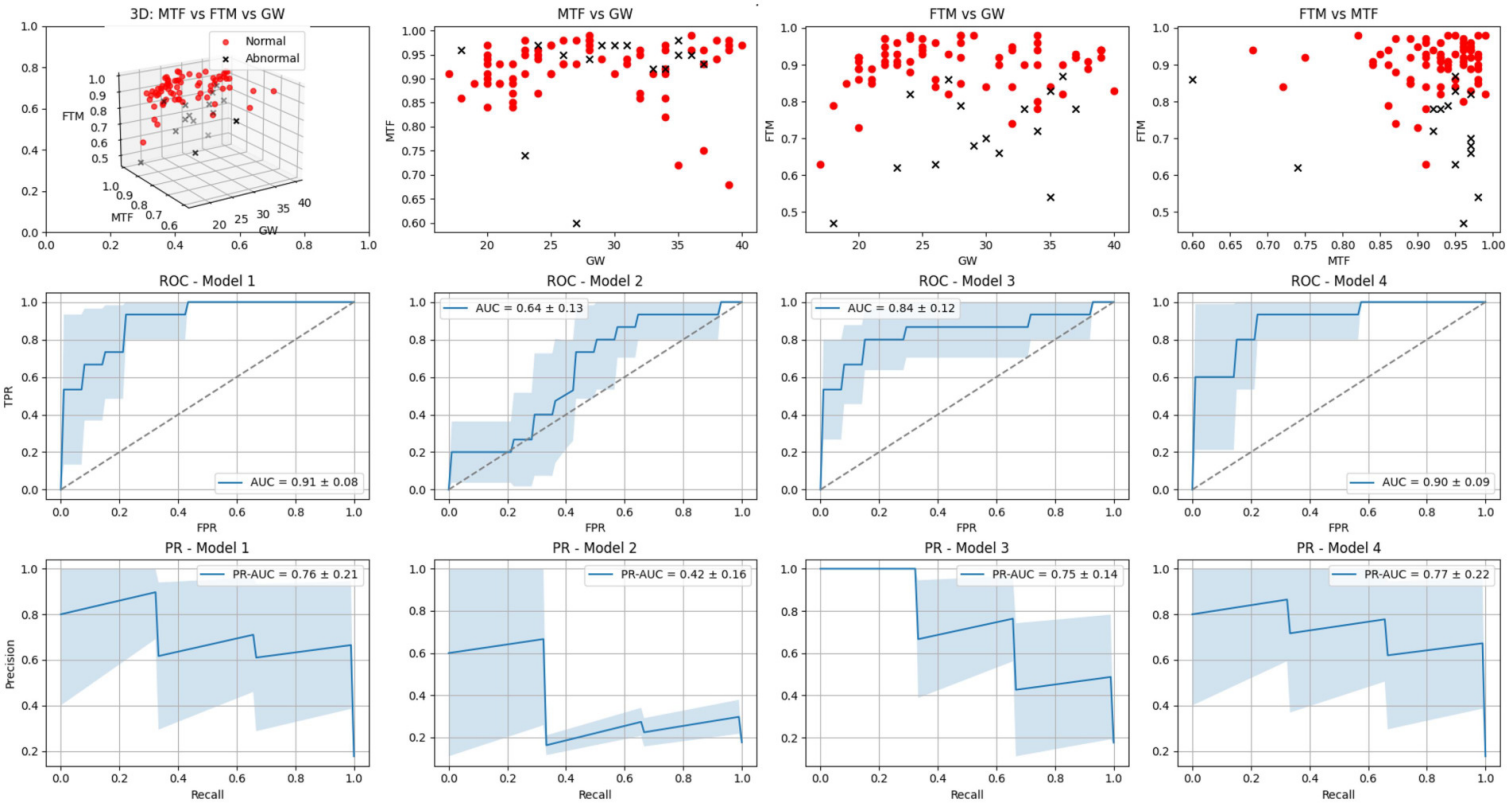
Feature visualizations (top row), corresponding receiver operating characteristic (ROC) curves (middle row), and precision–recall (PR) curves (bottom row) for four classification models under leave-one-subject-out cross-validation. ROC-AUC and PR-AUC values are reported as mean ± standard deviation.

To evaluate the potential of coupling features as clinical biomarkers for detecting abnormal fetal development, we performed a classification analysis focused on all normal and abnormal cases. We began by exploring the distribution of MTF and FTM coupling strengths across gestational age using four different visual perspectives. These included a 3D scatter plot of MTF and FTM coupling against gestational week, and separate 2D plots showing MTF vs. gestational week, FTM vs. gestational week, and MTF vs. FTM ignoring the gestational age information to mitigate bias due to the absence of abnormal cases in later gestation within our dataset. As shown in the top row of Figure 9, abnormal cases tended to cluster separately from normal cases, particularly in the FTM projection. This visual distinction suggests that FTM coupling, either alone or in conjunction with MTF coupling and/or gestational age, may have clinical relevance for detecting fetal abnormalities.

To validate this observation quantitatively, we trained four binary classification models using support vector machines (SVMs) (28). We employed the radial basis function (RBF) kernel (29), which is well-suited for capturing nonlinear relationships commonly found in biomedical datasets. Each model was configured to output class probabilities, allowing for the construction of receiver operating characteristic (ROC) curves to assess diagnostic performance (30). Due to the imbalance in class distribution (70 normal vs. 15 abnormal), class weights were incorporated into the SVM loss function to mitigate bias during training. Model evaluation was carried out using subject-wise LOSO cross-validation (25), and the resulting area under the ROC and PR curves scores are summarized in the bottom row of Figure 9.

The highest ROC-based classification performance was achieved when the model incorporated MTF coupling, FTM coupling, and gestational week as input features (ROC-AUC = 0.91 ± 0.08). A markedly lower performance was observed when only MTF coupling and gestational week were used (ROC-AUC = 0.64 ± 0.13). Interestingly, the model using only FTM coupling and gestational week achieved a substantially higher ROC-AUC of 0.84 ± 0.12, approaching the performance of the full-feature model. When MTF and FTM coupling were combined without gestational week, the classifier achieved ROC-AUC of 0.90 ± 0.09, indicating that bidirectional coupling features alone retain strong discriminative capability. In addition to ROC-AUC, precision–recall AUC (PR-AUC) was evaluated to account for class imbalance (70 normal vs. 15 abnormal cases). Notably, the coupling-only model (MTF + FTM) achieved the highest PR-AUC (0.77 ± 0.22), slightly exceeding the full-feature model (0.76 ± 0.21). This suggests that while gestational week provides marginal improvement in ROC-based discrimination, the intrinsic bidirectional coupling features alone are highly informative for identifying abnormal pregnancies under imbalanced conditions.

### 3.7 Negative control experiments

To further verify that the proposed deep coupling strength (DCS) reflects genuine physiological interaction rather than a generic prediction artifact, we conducted two negative-control experiments for both the maternal-to-fetal (MTF) and fetal-to-maternal (FTM) directions. In the first control, mismatched mother–fetus pairs were generated by pairing the input sequence from one subject with the target sequence from a different subject, thereby removing the true physiological relationship between the two signals. In the second control, temporally scrambled surrogate signals were generated by disrupting the original temporal order of the sequence while preserving its value range, which destroys the true temporal dependency structure. The DCS values obtained from these two control settings were then compared with those obtained from the true paired data.

**Figure 10:**
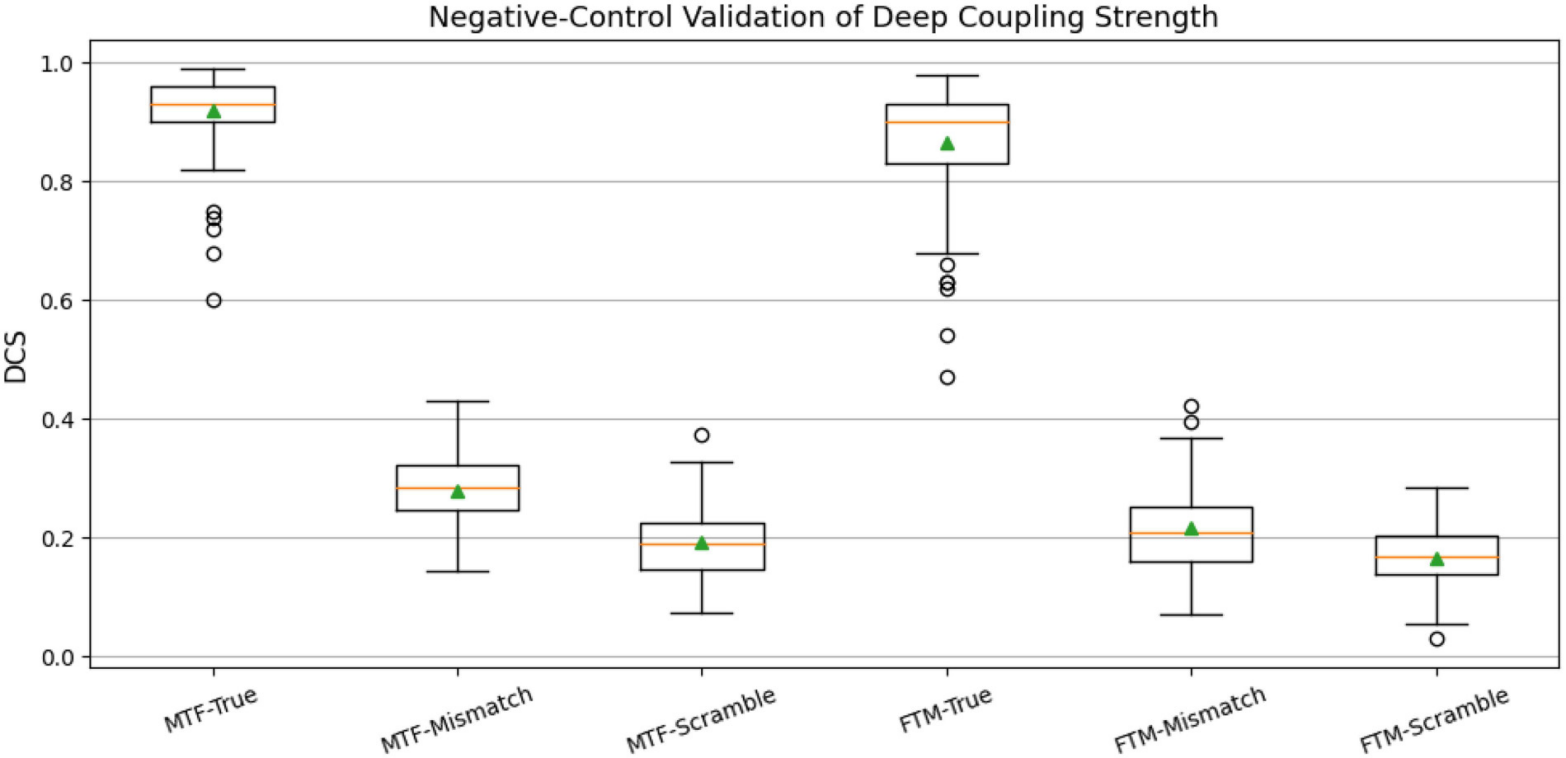
Negative-control validation of the proposed deep coupling strength (DCS) metric. The distributions of DCS obtained from the true paired data are compared with two negative-control settings: mismatched mother–fetus pairs across different subjects and temporally scrambled surrogate signals, for both the maternal-to-fetal (MTF) and fetal-to-maternal (FTM) directions.

Figure 10 shows that, in both MTF and FTM analyses, the true paired data consistently produced markedly higher DCS values than either the mismatched-pair or scrambled-signal controls. This clear reduction in DCS under the two negative-control conditions supports that the proposed metric captures meaningful maternal–fetal physiological coupling rather than trivial statistical similarity or model bias. Notably, both mismatch and scramble controls yielded low DCS distributions, confirming that preserving the true subject pairing and temporal structure is essential for maintaining the observed coupling strength.

## 4 Discussion

This study presents a deep learning framework for analyzing bidirectional maternal-fetal cardiac coupling based on heart rate variability (HRV) features extracted from electrocardiographic recordings. The analysis spans both MTF and FTM directions, accounting for gestational progression and pathological deviations. By integrating time-delay analysis, gestational trend evaluation, and classification modeling, we provide a comprehensive investigation into the dynamics and potential physiological relevance of maternal-fetal cardiac interactions.

The time-delay analysis (Figure 2) revealed a distinct asymmetry in the timing of coupling. MTF coupling strength peaked at a delay of approximately 3 s, suggesting a relatively prompt maternal influence on fetal heart dynamics. In contrast, FTM coupling reached its maximum at around 5 seconds, indicating a slower, potentially more indirect influence of fetal cardiac activity on maternal rhythms. This directional difference may reflect the physiological hierarchy between the maternal and fetal autonomic systems and emphasizes the importance of temporal modeling in understanding maternal-fetal coupling mechanisms. Importantly, this delay-dependent behavior also provides evidence that the observed coupling is not driven by trivial information leakage between signals. If fetal-to-maternal prediction were dominated by residual maternal components embedded in the fetal-derived input, one would expect the strongest apparent coupling to occur at (or very near) zero delay. Instead, the presence of distinct non-zero peak delays, and their directional asymmetry (MTF ≈ 3 s vs. FTM ≈ 5 s), supports a physiologically plausible time-lagged interaction rather than instantaneous circular inference.

From a methodological perspective, it is important to clarify that deep coupling strength does not quantify model performance in isolation but rather the extent to which one physiological system contains predictive information about the other under a constrained directional mapping. The reduction of DCS in abnormal cases, the directional delay asymmetry, and the differential discriminative power of FTM vs. MTF coupling collectively indicate that DCS behaves as a physiological dependency measure. If DCS merely reflected regression capacity, one would expect similar coupling strength across gestational conditions and no systematic directional structure. The observed deviations therefore support its interpretation as a dynamic inter-system coupling indicator.

When applied to a normal pregnancy case (Figure 3), the model demonstrated strong agreement between predicted and ground truth HRV features in both coupling directions. The coupling strength trajectories showed well-defined intervals of synchronization, which were further supported by Bland-Altman plots demonstrating narrow limits of agreement and minimal bias. These results indicate that the proposed model is capable of accurately capturing normal bidirectional cardiac interactions during gestation.

In contrast, analysis of an abnormal case involving intrauterine growth restriction (IUGR) at 37 weeks (Figure 4) revealed preserved accuracy in MTF coupling predictions but a significant drop in performance for FTM coupling. The reduced fetal-to-maternal influence may reflect disrupted autonomic signaling or fetal stress under pathological conditions. This observation aligns with prior evidence that fetal autonomic dysfunction is common in compromised pregnancies and suggests that impaired FTM coupling may be associated with abnormal fetal conditions.

Gestational trends in coupling strength further supported the model’s sensitivity to physiological changes over time. Figure 5 shows that MTF coupling strength remains relatively consistent across both normal and abnormal cases, which limits its utility for distinguishing abnormalities. However, as depicted in Figure 6, FTM coupling strength was consistently lower in abnormal cases compared to normal pregnancies, particularly during mid-to-late gestation. This divergence suggests that the fetal system’s contribution to maternal-fetal interaction becomes more pronounced as gestation progresses, and its impairment may represent an exploratory marker of developmental disruption or fetal distress rather than a clinically established biomarker.

In addition, the cohort-level regression analysis presented in Figure 7 provides a more detailed view of prediction errors across individual HRV features. For all five HRV metrics (mean R–R interval, SDNN, RMSSD, SD1, and SD2), the maternal-to-fetal (MTF) direction exhibits consistently low mean absolute error (MAE) with tightly distributed values for both normal and abnormal pregnancies. This indicates that the maternal physiological signals maintain stable predictive information about fetal HRV dynamics even under pathological conditions. In contrast, the fetal-to-maternal (FTM) direction shows a systematic increase in prediction error for abnormal pregnancies across all HRV features. While FTM errors remain relatively small in normal cases, abnormal cases demonstrate noticeably larger MAE distributions, reflecting a degradation in the predictive influence of fetal cardiac dynamics on maternal HRV. The consistency of this pattern across multiple HRV features reinforces the directional asymmetry observed throughout the study and supports the interpretation that reduced FTM coupling reflects impaired fetal-to-maternal physiological influence rather than instability of the regression framework. Importantly, the relatively low and stable MTF error distributions across the cohort confirm that the proposed deep learning model maintains reliable regression performance under both normal and pathological conditions.

Further examination of the MTF coupling distribution in abnormal cases reveals condition-specific deviations that provide additional physiological information. Patient 14, diagnosed with atrioventricular (AV) block and other congenital heart defects, exhibited the lowest MTF coupling strength, likely reflecting severely impaired cardiac conduction and limited fetal autonomic modulation of maternal rhythms. This observation has also been emphasized in (2). Similarly, patients 4 and 13 demonstrated markedly suppressed FTM coupling. In contrast, cases with arrhythmias such as fetal tachycardia and Wolff-Parkinson-White syndrome showed relatively preserved coupling values. These condition-specific observations should be interpreted cautiously due to the limited size and heterogeneity of the abnormal cohort, but they suggest that coupling metrics may warrant further investigation as exploratory phenotype-sensitive descriptors.

Here, it is important to note that the pathological cohort in this study is heterogeneous, including conditions with distinct etiologies. The observed reduction in FTM coupling strength should therefore not be interpreted as a single shared physiological mechanism but rather as a common emergent trend consistent with impaired autonomic regulation under multiple forms of fetal compromise. The present findings serve as exploratory evidence that warrants further validation in larger cohorts.

The classification analysis demonstrated that coupling metrics, particularly those involving FTM, show strong discriminative capability for detecting abnormal pregnancies. However, given the proof-of-concept nature of this study, the limited abnormal cohort, and the heterogeneity of the included pathologies, these findings should be interpreted as preliminary rather than as evidence of immediate clinical utility. Accordingly, bidirectional coupling should be viewed here as a promising exploratory signal representation for future prenatal risk stratification studies rather than a validated clinical biomarker.

A key limitation of this study is the relatively small number of abnormal subjects, which may introduce small-sample bias and restrict the statistical power of the abnormality analysis. In addition, the normal recordings are longer than the abnormal recordings, leading to an imbalance in the number of available training windows per subject during model fine-tuning. In the present framework, this imbalance was only partially mitigated by aggregating the deep coupling strength (DCS) at the subject level, ensuring that each subject contributes a single value to the cohort-level analysis, and by using class-weighted learning during the classification stage. However, no explicit balanced per-subject or per-class window sampling strategy was applied. Therefore, the effect of training imbalance may not be fully eliminated and should be considered a limitation of this proof-of-concept study. Future work will investigate balanced sampling strategies and larger, more balanced multi-center datasets to assess the robustness of the proposed framework.

Altogether, the results of this study offer new insights into the bidirectional and time-sensitive nature of maternal-fetal cardiac coupling. The observed asymmetries, gestational patterns, and pathology-specific deviations provide a strong physiological and computational basis for coupling-based analysis. At this stage, these findings should be interpreted as proof-of-concept evidence rather than as a basis for direct clinical application. The ability of the proposed model to detect subtle impairments in fetal influence motivates further validation in larger and more homogeneous cohorts to evaluate its potential role in non-invasive prenatal monitoring.

## 5 Conclusion

This study establishes a foundational framework for analyzing bidirectional maternal-to-fetal and fetal-to-maternal cardiac coupling using deep learning. By leveraging an advanced transformer-based deep network, we demonstrate the feasibility of accurately predicting HRV features in both directions, revealing physiological interactions between maternal and fetal cardiac systems. The results highlight the reciprocal influence between maternal and fetal cardiac dynamics, offering a potential pathway for integrating data-driven tools into prenatal care. Nevertheless, these findings should be interpreted as proof-of-concept evidence, given the limited size and heterogeneity of the abnormal cohort. Future work will focus on validation using larger and more homogeneous datasets to further assess the potential of this approach for non-invasive prenatal monitoring.

## Data Availability

The data analyzed in this study are subject to the following licenses/restrictions: Data are available upon a proper request by contacting Dr. Yoshiyuki Kasahara. Requests to access these datasets should be directed to Dr. Yoshiyuki Kasahara, kasa@med.tohoku.ac.jp.
